# Kimura’s Disease – An Unusual Presentation 

**Published:** 2016-05

**Authors:** Praveer Kumar Banerjee, Abhineet Jain, Manjunath D

**Affiliations:** 1 *Department of Otorhinolaryngology, JLN Hospital & RC, Sector-9, Bhilai-490009, ChattisgarhIndia.*; 2 *Department of Otorhinolaryngology, Karnatka Institute of Medical Sciences, Hubli-(karnatka), India. *

**Keywords:** Kimura’s, Lymphadenopathy, Subcutaneous mass, Candida, Eosinophilia, IgE

## Abstract

**Introduction:**

Kimura’s disease is a rare chronic inflammatory disease of unknown etiology, presenting as painless subcutaneous nodules with lymphadenopathy and peripheral eosinophilia, mainly disturbing the head and neck region. It mainly affects Asian males in their 2^nd^ to 4^th^ decade of life. One such case of Kimura’s disease, which is uncommon in Indian natives, is reported.

**Case Report:**

A male patient presented with an insidious onset of a progressive painless disfiguring swelling over his nose since four years, which was associated with nasal obstruction and post aural swelling with a history of an inconclusive incisional biopsy. Clinical examination showed a bilobed subcutaneous swelling present over the nose and a collapsed nasal valve area on anterior rhinoscopy. FNAC was non-diagnostic and CT scan showed a mildly enhancing mass lesion over the external nose. Complete surgical excision was performed. Diagnosis was confirmed upon postoperative histopathology. During his 2nd week follow up, the patient had a small nasal recurrence, which was treated medically with oral steroids, cetirizine, and pentoxyphylline for 4 weeks. The patient was disease free for 6 months.

**Conclusion:**

Kimura’s disease, although difficult to diagnosis clinically, should be considered in the differential diagnosis of patients who have a primary lymphadenopathy with eosinophilia with or without subcutaneous nodules. It should be investigated accordingly as the disease has an indolent course and good prognosis.

## Introduction

Kimura’s disease is a chronic inflammatory condition of unknown etiology presenting as multiple painless solitary subcutaneous nodules localized mostly in the region of the head and neck with coexisting lymphadenopathy and peripheral eosinophilia. Kimura’s disease is limited to the skin, lymph nodes, and salivary glands. Renal involvement is its only systemic manifestation. This rare condition is found almost exclusively in Asian individuals in their 2nd to 4th decade of life and mostly affects males (70–80%) (1,2). Management of this disease is personalized due to lack of consensus. In addition, a conservative approach is best suited for treatment.

## Case Report

**Fig 1:**
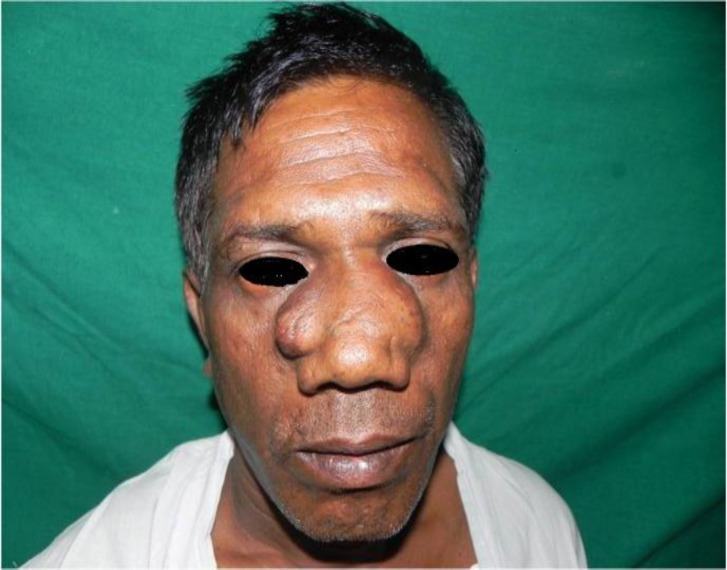
Pre op. Picture of patient with nasal mass

A 54-year-old male resident of Bhilai, India presented with complaints of an insidious onset of a gradually progressive swelling of the nose since 4 years and a similar swelling behind his right ear since 1 year (Fig.1). 

**Fig 2:**
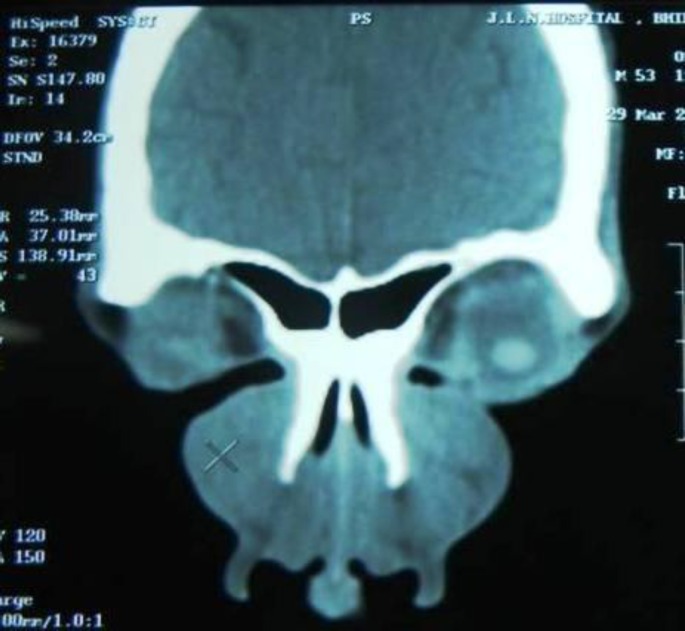
CT scan showing mild enhancing mass over nasal bones with no erosion of bones

He had no associated constitutional symptoms and no nasal or ear discharge. Upon physical examination, a 5×4 cm swelling was observed at the nasal dorsum and a 3×2 cm oval-shaped swelling was observed in the right post aural region. This swelling was non-tender, firm to soft and non-compressible. The skin over the swelling was normal. The FNAC yielded bloody aspirate which was inconclusive. Hematological investigations revealed eosinophilia (23%) and elevated serum IgE (210 U/ml). CT scan revealed a mildly enhancing mass present over the nasal bones (Fig.2).

**Fig 3:**
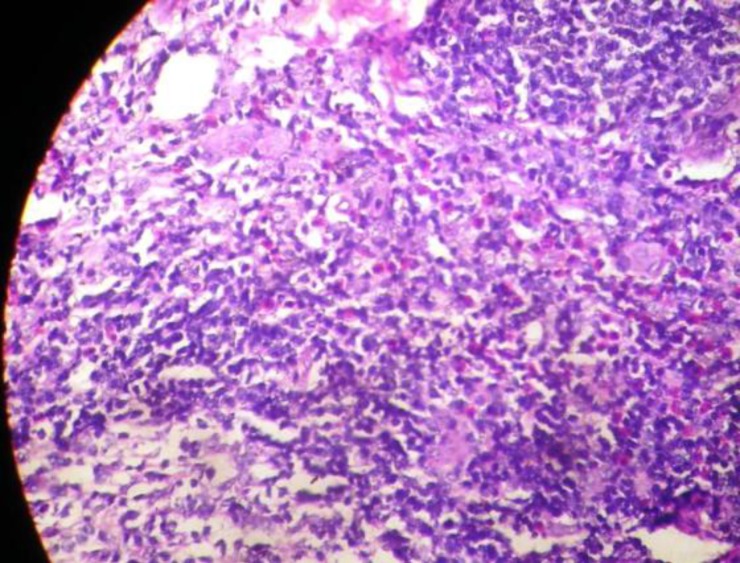
HPE showing plumb like endothelial cells, eosinophils and interstitial fibrosis

Surgical excision was performed and histopathology revealed plumb like epitheloid endothelial cells, eosinophilia, and interstitial fibrosis, which are all features suggestive of Kimura's disease (Fig.3). During his 2nd week follow up, the patient had a small nasal recurrence. It was treated medically with oral Prednisolone 40mg/day for 2 weeks, which was then gradually tapered to 10mg/day until the 4th week, oral Cetirizine 10mg OD, and oral Pentoxyphylline 400mg TDS for 4 weeks, to reduce immunity and innate immunity. The patient was disease free for 6 months.

## Discussion

Kimura's disease is an unusual condition of uncertain etiology. First described in 1937 and later popularized in 1947 by Kimura and his associates, it is a benign disease which involves subcutaneous tissues (preauricular, submandibular), the major salivary gland, and lymph nodes mainly in the head and neck area (3). However, other sites such as the eyelids, orbit, oral cavity, groin, trunk, and limbs may also be involved. China and Japan are endemic countries, although sporadic cases are described elsewhere (4). The most popular theory is that of Candida acting as a source of persistent antigenemia, although neither hyphae nor spores have been isolated. Kimura’s disease may affect the kidneys in up to 60% of patients, presenting as all types of glomerulonephritis or as nephritic syndrome (12%) (2,5). Hypereosinophilia and elevated serum IgE are found in Kimura’s disease as well.

Diagnosis through FNAC is misleading and can easily be mistaken for a malignant disorder. Diagnosis is therefore only established through histopathological examination. T-cell lymphoma, Kaposi Sarcoma, Hodgkin’s disease, and angio- lymphoid hyperplasia with eosinophilia are potential differential diagnoses (Churia et al, 1997). Differential diagnosis between Kimura’s disease (KD) and angiolymphoid hyperplasia with eosinophilia (ALHE) has been a challenge for a long time. In contrast to ALHE, in Kimura's disease, germinal centers are destroyed due to heavy infiltration of eosinophils and absence of vacuolated endothelial cells. Immunofluorescence tests show heavy IgE deposits and variable amounts of IgG, IgM, and fibrinogen (1,6). However, these tests were not performed in our cases. 

There is no consensus on the management of Kimura’s disease; however, a conservative approach may be sufficient with the use of other modalities of treatment. Conservative treatment includes oral steroids; however, the lesions usually get enlarged again when steroid treatment is terminated. Thus successful treatment is mainly reassured by a constant low dose of steroids (2,6,7,8,). Surgery and subsequent steroid treatment are proposed as an alternative treatment (6). Radiation therapy in doses of 20–30 Gy is useful to control lesions that relapse after surgery or are unresponsive to steroids (9). Other treatment methods include Retinoids, monoclonal antibodies (Imatinib), antiallergic drugs (Cetirizine), and Pentoxifylline (10), with variable effect. 

This case is exceptional as - 1) It is a sporadic case found in non-Orientals. 2) It shows involvement of the nose with no lymphadenopathy 3) Inconclusive FNAC is seen and histological diagnosis led us to do retrospective investigations and 4) Grossly elevated IgE levels are seen. In addition, there was marked eosinophilia; however, the diagnosis was not suspected as tropical eosinophilia is not uncommon in this country.

## Conclusion

Kimura’s disease, although difficult to diagnose clinically, should be considered in the differential diagnosis in patients who show primary lymphadenopathy with eosinophilia with or without subcutaneous nodules. It should be investigated accordingly as the disease has an indolent course and good prognosis.
